# Utility of Fluorodeoxyglucose Positron Emission Tomography/Computed Tomography (FDG-PET/CT) Imaging for Evaluating Treatment Efficacy in Mycobacterium avium Complex Pulmonary Disease

**DOI:** 10.7759/cureus.79891

**Published:** 2025-03-01

**Authors:** Takahiro Takazono, Naoki Hosogaya, Reiko Ideguchi, Masataka Yoshida, Kazuaki Takeda, Shotaro Ide, Naoki Iwanaga, Ryo Toya, Takashi Kudo, Koichi Izumikawa, Katsunori Yanagihara, Hiroshi Mukae

**Affiliations:** 1 Department of Respiratory Medicine, Nagasaki University Hospital, Nagasaki, JPN; 2 Department of Infectious Diseases, Nagasaki University Graduate School of Biomedical Sciences, Nagasaki, JPN; 3 Clinical Research Center, Nagasaki University Hospital, Nagasaki, JPN; 4 Department of Radioisotope Medicine, Atomic Bomb Disease Institute, Nagasaki University, Nagasaki, JPN; 5 Infectious Diseases Experts Training Center, Nagasaki University Hospital, Nagasaki, JPN; 6 Department of Radiation Oncology, Nagasaki University Hospital, Nagasaki, JPN; 7 Department of Infectious Diseases, Nagasaki University Hospital, Nagasaki, JPN; 8 Department of Laboratory Medicine, Nagasaki University Hospital, Nagasaki, JPN; 9 Department of Respiratory Medicine, Nagasaki University Graduate School of Biomedical Sciences, Nagasaki, JPN

**Keywords:** disease activity assessment, fdg-pet/ct, mac-pd, nontuberculous mycobacteriosis, prospective study

## Abstract

Introduction

*Mycobacterium avium* complex pulmonary disease (MAC-PD) necessitates prolonged treatment. However, determining the appropriate time to conclude treatment is challenging because of the lack of indices for evaluating disease activity. Fluorodeoxyglucose positron emission tomography/computed tomography (FDG-PET/CT) can be used to visualize inflammatory sites and is hypothesized to indicate treatment efficacy in inflammatory diseases. Therefore, we conducted this single-arm, open-label, interventional study to investigate the utility of FDG-PET/CT imaging as an indicator of treatment efficacy by analyzing the correlation between pre- and post-treatment FDG-PET/CT imaging and treatment efficacy in MAC-PD.

Methods

FDG-PET/CT scans were performed before and 52 weeks after the initiation of MAC-PD treatment to assess the utility of FDG-PET/CT as an indicator of treatment efficacy. The primary endpoint was the association between treatment efficacy/culture-negative conversion and the standardized uptake value (SUV) max, SUV peak, target/background ratio (TBR), metabolic tumor volume (MTV), and total lesion glycolysis (TLG).

Results

Ten patients were enrolled after obtaining informed consent, and comparative evaluation after one year of treatment was feasible in nine patients. Based on predefined comprehensive clinical endpoints (symptoms, imaging, and inflammatory findings), five cases were deemed effective, and four cases were deemed ineffective. The effective cases exhibited a significantly lower SUV peak and TBR in the pre- and post-treatment ratios than the ineffective cases. However, no statistically significant associations were observed between these indices and culture-negative conversions.

Conclusion

These findings suggest that the pre- and post-treatment ratios of SUV peak and TBR may be valuable for evaluating disease activity in patients with MAC-PD.

## Introduction

Non-tuberculous mycobacteria (NTM) are organisms found in environmental water, soil, and animal hosts and are typically transmitted to humans via the respiratory tract. In Japan, approximately 90% of NTM infections are caused by *Mycobacterium avium* complex (MAC), which induces slowly progressive chronic inflammation [1]. The incidence of NTM and its associated mortality are increasing [2,3]. However, effective antimicrobial agents are limited. Although the American Thoracic Society/European Respiratory Society/European Society of Clinical Microbiology and Infectious Diseases/Infectious Diseases Society of America (ATS/ERS/ESCMID/IDSA) guidelines recommend long-term treatment, there are no specific biomarkers to assess disease activity, and the decision to terminate treatment is often challenging [4]. Furthermore, long-term antimicrobial therapy may lead to adverse events, and appropriate assessment of disease activity remains a significant challenge in the management of NTM.

18F-fluorodeoxyglucose positron emission tomography (FDG-PET) is utilized not only for localizing malignant tumors and detecting metastases but also for visualizing inflammatory sites and the localization and diagnosis of large-vessel vasculitis [5]. FDG-PET has also been used to determine disease activity. For instance, it has been proposed as a method for assessing the efficacy of treatment for malignant lymphoma [6,7]. In the field of infectious diseases, FDG-PET has demonstrated utility as an indicator of therapeutic efficacy in experimental pulmonary tuberculosis infection in rabbits [8] and has been observed to accumulate in the foci of MAC pulmonary disease (PD) and chronic pulmonary aspergillosis (CPA) [9,10]. The efficacy of FDG-PET in evaluating disease activity and severity in multidrug-resistant TB and NTM has been investigated [11-13], and the potential of FDG-PET for determining treatment efficacy has garnered attention. However, no prospective studies have evaluated the utility of FDG-PET/CT before and after treatment.

Therefore, we conducted this single-arm, open-label, interventional study to investigate the utility of FDG-PET/CT imaging as an indicator of treatment efficacy by analyzing the correlation between pre- and post-treatment FDG-PET/CT imaging and treatment efficacy for MAC-PD.

## Materials and methods

Research design and ethical considerations

This was a physician-led, single-arm, open-label, exploratory intervention. This interventional study was approved by the Nagasaki University Clinical Research Review Committee (approval no. CRB7180001) (permit no. CRB19-014) and was conducted at Nagasaki University Hospital after registration in the Japan Registry of Clinical Trials (jRCTs071190035), a database maintained by the Ministry of Health, Labor, and Welfare of Japan (https://jrct.niph.go.jp/).

The study was conducted in accordance with the ethical principles of the Declaration of Helsinki [14]. The study participants were included after obtaining written consent.

Inclusion and exclusion criteria

Patients who were not treated with antimicrobial agents and were at least 20 years of age and diagnosed with pulmonary MAC disease according to the ATS/American Society of Infectious Diseases Guidelines 2007 [15] were included.

The exclusion criteria are as follows: patients with poor glycemic control (fasting blood glucose level >200 mg/dL), patients with cystic fibrosis, patients with active respiratory tract infections other than MAC-PDs, patients with lung cancer at the time of consent, and pregnant women or women who may become pregnant.

Methods

The study began in December 2019 with a research intervention of FDG-PET/CT before (0 weeks) and 52 weeks after treatment initiation for MAC-PD. The attending physician selected the treatment for MAC-PD according to the latest guidelines. Continuation of treatment after the second FDG-PET/CT scan at 52 weeks or discontinuation of treatment owing to side effects was left to the discretion of the attending physician.

Criteria for efficacy judgment

In MAC-PD, treatment efficacy is mainly evaluated by the negative conversion of sputum culture; however, in some cases, sputum cannot be expectorated, making evaluation difficult. The culture-negative conversion was defined as at least one negative result in MGIT media culture at the predefined timing of evaluation. Furthermore, there are no established criteria for clinical efficacy. Therefore, in this study, we applied the clinical efficacy criteria for pneumonia from the Clinical Evaluation of New Antimicrobial Agents for Respiratory Infections (Second Edition) [16] and modified it according to the pathogenesis of MAC-PD. We categorized patients as follows: "effective," "ineffective" (if the criteria for effective were not met), and "not judged" (if the information on symptoms, physical findings, temperature, white blood cells, and C-reactive protein (CRP) were missing).

The criteria for "effective" encompassed the fulfillment of criterion a, the fulfillment of either criterion b or c, and the absence of deterioration in the remaining items. Criterion a comprised the following: resolution or improvement of the clinical symptoms of MAC-PD, with particular attention to symptoms such as fever, cough, sputum volume, hemosputum, dyspnea, and others. This required improvement in at least one symptom or finding. For participants presenting with fever (38°C or higher) at the time of enrollment, fever reduction was mandatory. A decrease in temperature from that recorded at the time of registration, even if it remained at or above 37.0℃, was considered an improvement in fever. Criterion b involved the improvement of abnormal findings on chest imaging, based on the size and density of the foci. Criterion c pertained to the improvement of inflammatory findings, specifically the reduction of white blood cell count to 9,000/mm³ or less, a decrease in CRP from the highest level, and the absence of deterioration in any of these conditions.

The principal investigator and all the sub-investigators for this study were respiratory medicine specialists and infectious disease specialists.

Image analysis

The standardized uptake value (SUV) max, SUV peak, target/background ratio (TBR), metabolic tumor volume (MTV), and total lesion glycolysis (TLG) were evaluated as semi-quantitative indices of FDG accumulation; the threshold for MTV was established at SUV max 2.5, TLG was calculated as the lesion SUV mean multiplied by MTV, and TBR as the lesion peak divided by the normal lung mean. Metavol, an open-source software package, was used for the analysis. SUV max is a frequently utilized parameter in the evaluation of FDG-PET. Furthermore, volume indices such as MTV and TLG were employed, as it was deemed necessary to assess the activity of the entire lesion in MAC-PD that can manifest multiple lesions in both lungs. MTV and TLG are also utilized to determine the efficacy of treatment for malignant neoplasms, and their application in the evaluation of inflammatory diseases was considered appropriate. The detailed methodology for the analysis is provided in Appendix A.

Endpoints

The primary endpoint was the association between treatment efficacy/culture-negative conversion and SUV max, SUV peak, target/background ratio (TBR), MTV, and total lesion glycolysis (TLG). The secondary endpoints were the presence or absence of antimicrobial treatment between weeks 52 and 76 of treatment, whether treatment was resumed in patients who had completed treatment, and the relationship between treatment efficacy and the chronic obstructive pulmonary disease (COPD) assessment test (CAT), body mass index (BMI), and erythrocyte sedimentation rate (ESR).

Basis for setting the number of cases

As this was an exploratory study, the target number of cases was set at 10 cases of pulmonary MAC disease based on feasibility.

Statistical analysis

The ratios of FDG-PET/CT SUV max, SUV peak, TBR, MTV, and TLG before and 52 weeks after the start of treatment were compared between “effective” and “ineffective” groups, and the Mann-Whitney test was performed. Receiver operating characteristic (ROC) curves for the clinical efficacy of each test were generated, and the AUCs were calculated. Cutoff values were determined by the Youden index. GraphPad Prism 9 (GraphPad Software Inc., USA) and JMP Pro 18 (SAS Institute, Japan) were used for graph generation and statistical analyses.

## Results

The characteristics of the patients with MAC-PD, the results of each FDG-PET/CT scan, and treatment efficacy are summarized in Table 1. The mean age was 72.5 years (range: 53-85 years). Six and four patients had *M. avium* and *M. intracellulare *infections, respectively. The underlying diseases were rheumatoid arthritis, aortic stenosis, and hypertension in one case each. One of these patients (Case 2) discontinued MAC-PD treatment and hospital visits at the patient's request and consequently could not be evaluated following the initiation of treatment. Two patients completed the treatment 52 weeks after PET/CT. No adverse events associated with FDG-PET/CT were observed. Throughout the study period, participants did not experience complications from other infectious diseases such as pneumonia.

**Table 1:** Case summary and results of FDG-PET/CT. NB: nodular bronchiectasis; CNB: cavitary nodular bronchiectasis; SUV: standardized uptake value; TBR: target/background ratio; MTV: metabolic tumor volume; TLG: total lesion glycolysis; RECAM: rifampicin+ethambutol+clarithromycin; CAM: clarithromycin; RFP: rifampicin; EB: ethambutol; LVFX: levofloxacin; MFLX: moxifloxacin; AE: adverse effect; FDG-PET/CT: fluorodeoxyglucose positron emission tomography/computed tomography

Case no.	Age	Sex	Type of CT finding	Strain of MAC	SUV max (Bq/g) 0/52 weeks	SUV peek (Bq/g) 0/52 weeks	TBR 0/52 weeks	MTV (ml) 0/52 weeks	TLG (ml) 0/52 weeks	Culture negative conversion	Clinical efficacy criterion a after 52 weeks	Clinical efficacy criterion b after 52 weeks	Clinical efficacy criterion c after 52 weeks	Total clinical effectiveness after 52 weeks	Treatment continuation at 76 weeks	Clinical effectiveness after 76 weeks	Treatment regimen
1	68	M	NB	M. avium	2.45/2.5	1.64/2.03	4.41/5.24	0/0	0/0	No	No	No	No change	No	Yes	No	RECAM→CAM, RFP, LVFX due to AE of EB
2	59	F	NB	M. avium	5.58/-	3.22/-	13.7/-	1.05/-	3.57/-	-	-	-	-	-	-	-	Started with RECAM, but stopped by patient's requirement
3	75	M	NB	M. intracellulare	1.97/4.2	1.57/3.9	4.98/9.88	0/6.17	0/19.43	Yes	No change	No	No change	No	Yes	No	RECAM
4	85	F	CNB	M. avium	6.74/6.41	5.57/4.81	13.11/13.5	70.22/29.06	243.88/94.43	No	No change	No	No change	No	Yes	No	RECAM→CAM, MFLX due to AE of RFP and EB
5	70	F	NB	M. intracellulare	12.19/13.95	9.43/11.55	29.92/37.15	111.97/70.72	529.95/336.11	No	No	No	No change	No	Yes	No	RECAM→CAM, RFP, LVFX due to AE of EB
6	84	F	NB	M. avium	2.85/3.18	2.28/2.37	5.76/6.66	0.25/0.26	0.67/0.7	N/A	Yes	Yes	No change	Yes	Yes	Yes	RECAM
7	74	M	NB	M. avium	4.55/1.93	3.22/0.77	10.02/3.22	2.84/0	8.7/0	Yes	Yes	Yes	Yes	Yes	Finished	Recurrence	RECAM
8	53	F	NB	M. intracellulare	16.69/1.22	12.03/1.01	40.63/3.76	60.81/0	236.4/0	Yes	Yes	Yes	No change	Yes	Finished	No recurrence	RECAM
9	79	F	NB	M. intracellulare	3.63/1.61	2.71/1.21	4.71/3.2	4.13/0	11.57/0	Yes	Yes	Yes	Yes	Yes	Yes	Yes	RECAM
10	78	M	NB	M. avium	13.05/1.57	4.82/1.04	23.62/3.94	1.93/0	8.06/0	Yes	Yes	Yes	No change	Yes	Yes	Yes	RECAM

**Figure 1:**
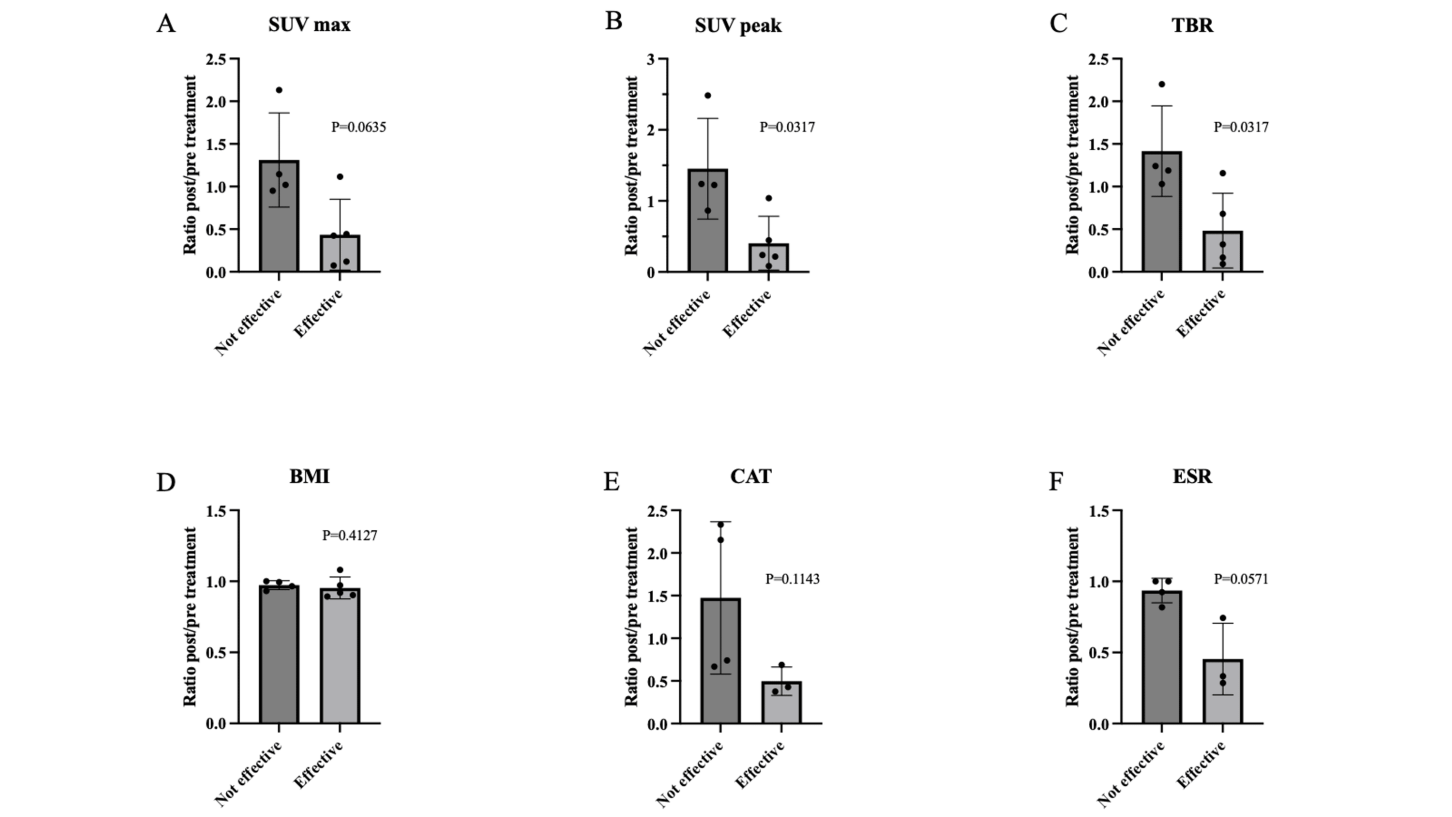
Comparison of pre- and post-treatment ratios of each test between the effective and ineffective cases. The figure represents SUV max (A), SUV peak (B), TBR (C), BMI (D), CAT (E), and ESR (F). The pre- and post-treatment ratios of the SUV peak and TBR were significantly lower in the effective cases. The comparison was conducted using the Mann-Whitney test. A p-value of less than 0.05 was considered statistically significant. Two missing CAT and ESR values are excluded from the analysis. SUV max represents the highest standardized uptake value within the region of interest. SUV peak is the average SUV within a small, typically spherical volume (e.g., 1 cm³) centered around the region with the highest uptake. TBR is the ratio of the SUV in the region to the SUV of the normal lung mean. SUV: standardized uptake value; TBR: target-to-background ratio; BMI: body mass index; CAT: chronic obstructive pulmonary disease assessment test; ESR: erythrocyte sedimentation rate

**Figure 2:**
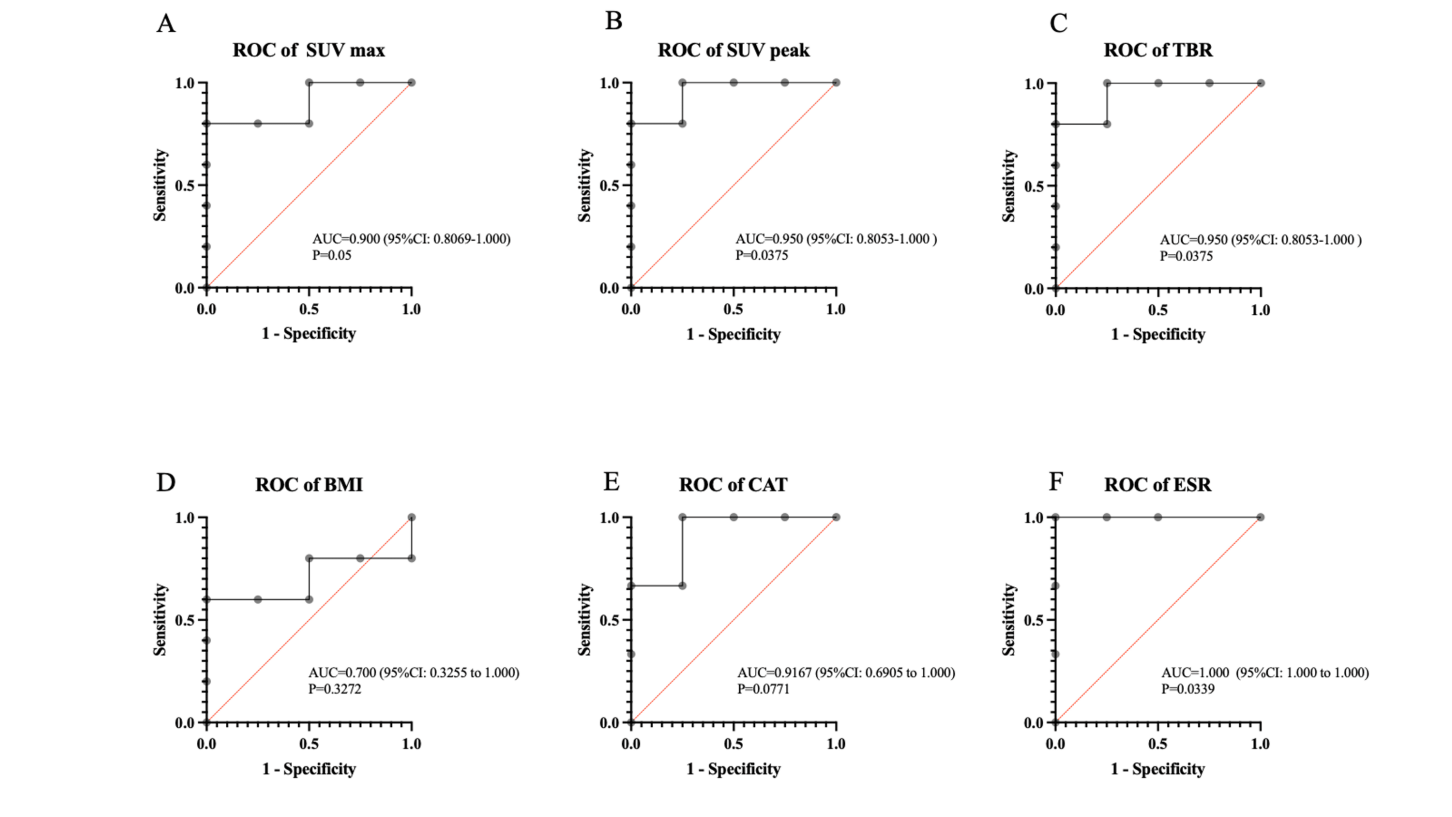
Receiver operating curves (ROCs) of the pre- and post-treatment ratios of each test for evaluation of treatment effectiveness. The cutoff values of pre- and post-treatment ratios for SUV max (A), SUV peak (B), TBR (C), BMI (D), CAT (E), and ESR (F) were 0.443 (sensitivity 80%, specificity 100%), 0.446 (sensitivity 80%, specificity 100%), 0.679 (sensitivity 80%, specificity 100%), 0.919 (sensitivity 60%, specificity 100%), 0.687 (sensitivity 75%, specificity 100%), and 0.74 (sensitivity 100%, specificity 100%), respectively. SUV: standardized uptake value; TBR: target-to-background ratio; BMI: body mass index; CAT: chronic obstructive pulmonary disease assessment test; ESR: erythrocyte sedimentation rate. Two missing CAT and ESR values are excluded from the analysis. SUV max represents the highest standardized uptake value within the region of interest. SUV peak is the average SUV within a small, typically spherical volume (e.g., 1 cm³) centered around the region with the highest uptake. TBR is the ratio of the SUV in the region to the SUV of the normal lung mean.

Five cases were determined to be "effective" based on the aforementioned criteria, while four cases were deemed "ineffective.” The pre- and post-treatment MTV and TLG ratios were not analyzed because of the inability to calculate these ratios in both groups as some patients exhibited values below the predefined threshold (SUV max). The pre- and post-treatment ratios of the SUV peak and TBR were significantly lower in the effective cases (Figure 1). The ROC curves for each test are shown in Figure 2. The results were statistically significant for SUV max, SUV peak, TBR, and ESR. The cutoff values of pre- and post-treatment ratios for SUV max, SUV peak, TBR, BMI, CAT, and ESR were 0.443 (sensitivity 80%, specificity 100%), 0.446 (sensitivity 80%, specificity 100%), 0.679 (sensitivity 80%, specificity 100%), 0.919 (sensitivity 60%, specificity 100%), 0.687 (sensitivity 75%, specificity 100%), and 0.74 (sensitivity 100%, specificity 100%), respectively.

**Figure 3:**
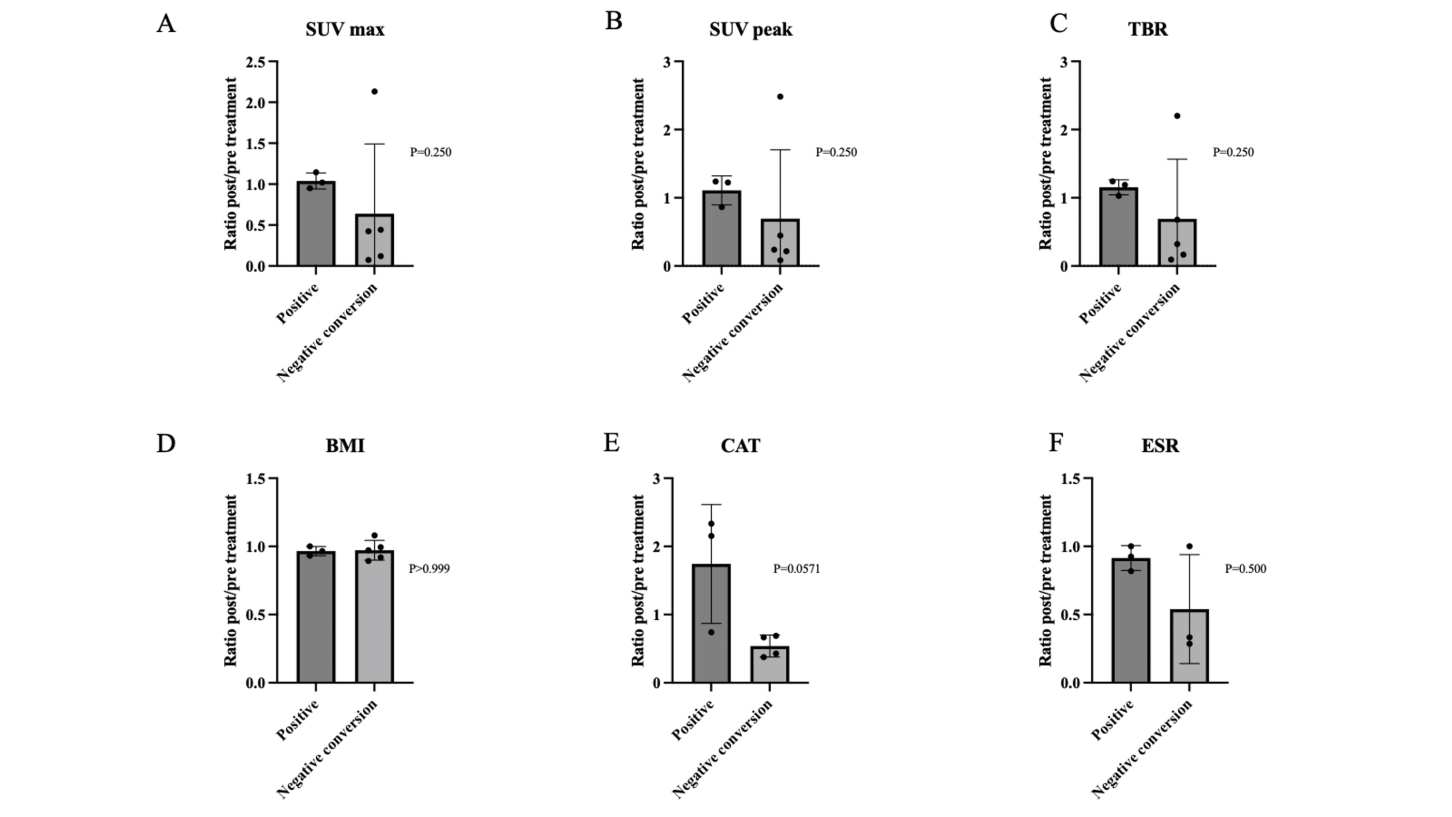
Comparison of pre- and post-treatment ratios of each test between the culture-negative conversion cases and culture-positive cases at 52 weeks after treatment initiation. The figure represents SUV max (A), SUV peak (B), TBR (C), BMI (D), CAT (E), and ESR (F). No statistically significant association is observed between these indices and culture-negative conversions. The comparison was conducted using the Mann-Whitney test. A p-value of less than 0.05 was considered statistically significant. SUV max represents the highest standardized uptake value within the region of interest. SUV peak is the average SUV within a small, typically spherical volume (e.g., 1 cm³) centered around the region with the highest uptake. TBR is the ratio of the SUV in the region to the SUV of the normal lung mean. SUV: standardized uptake value; TBR: target-to-background ratio; BMI: body mass index; CAT: chronic obstructive pulmonary disease assessment test; ESR: erythrocyte sedimentation rate

At 52 weeks after the initiation of antimycobacterial therapy, sputum culture results became negative in five patients, remained positive in three patients, and one patient experienced difficulty in expectorating sputum. The negative conversion of sputum culture was analyzed in relation to these PET/CT indices as well as BMI, CAT, and ESR (Figure 3). However, no statistically significant associations were observed between these indices and culture-negative conversions.

**Figure 4:**
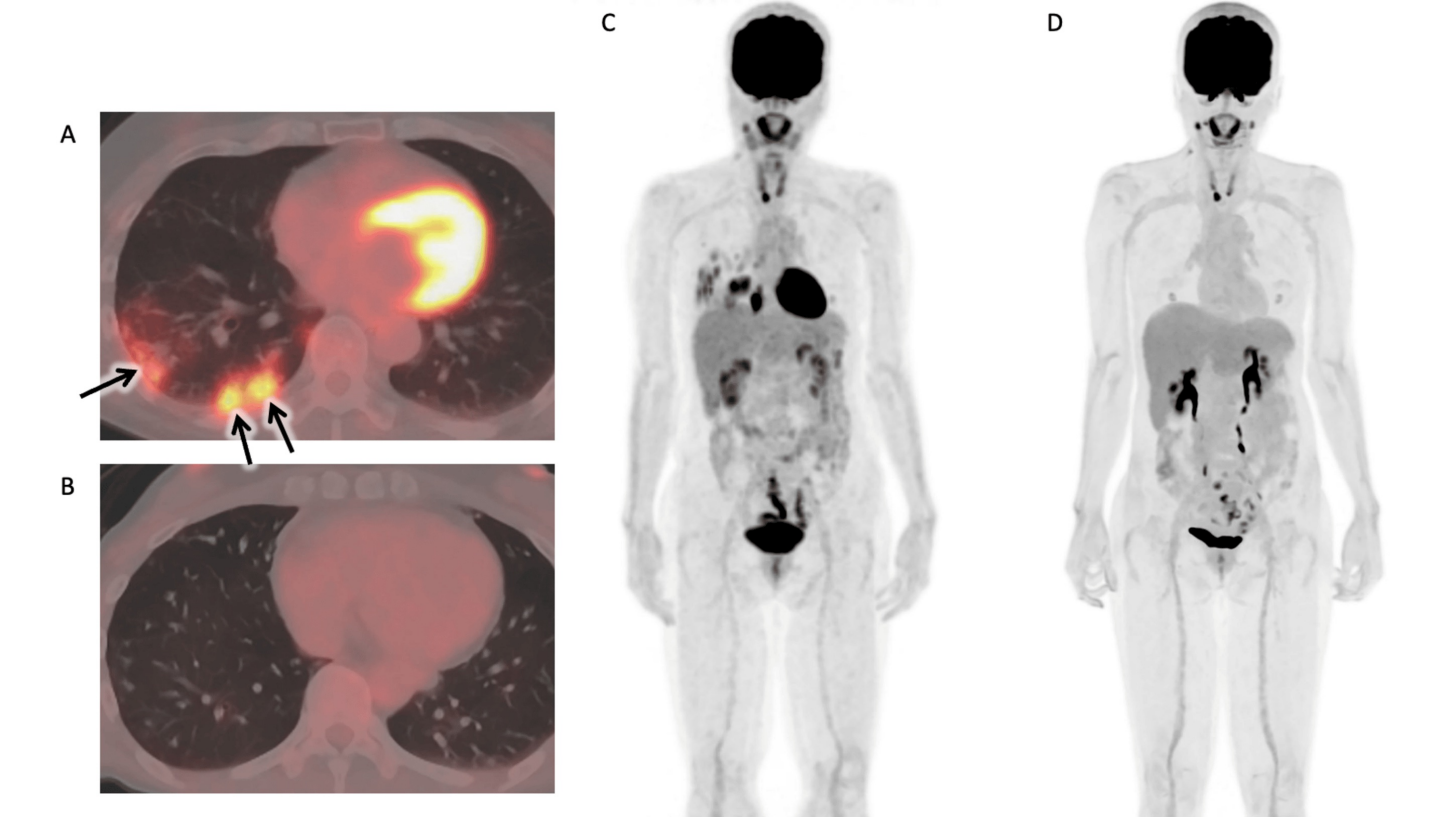
FDG-PET/CT images of a representative case (Case 8) who responded to anti-mycobacterial therapy. Prior to treatment initiation, high FDG uptake is observed in multiple nodules (black arrows) within the right lower lobe (A: axial image; C: coronal image). At 52 weeks post-treatment, FDG uptake exhibited a near-complete reduction (B: axial image; D: coronal image). FDG-PET/CT: fluorodeoxyglucose positron emission tomography/computed tomography

Figure 4 presents the FDG-PET/CT images of a representative case (Case 8) that exhibited a positive response to treatment. Pre-treatment PET/CT images demonstrated elevated FDG accumulation in multiple nodular shadows within the right lower lobe, whereas PET/CT images obtained 52 weeks after initiation showed minimal accumulation (SUV peak=1.01). The patient completed the treatment regimen at one year, and no recurrence was observed during the subsequent follow-up period.

## Discussion

In this study, we prospectively evaluated the usefulness of FDG-PET/CT for determining treatment efficacy in patients with MAC-PD. These results suggest that the pre- and post-treatment ratios of the SUV peak may be particularly useful for determining the efficacy of treatment by using the provided cutoff value. It has recently been reported that changes in SUV max, SUV peak, and TLG before and after six months of treatment for CPA were associated with the disease status of CPA. The results of this study on CPA are consistent with those of our study [17]. ESR and CAT may also be useful for evaluating disease activity in patients with MAC-PD; however, these tests can be affected by other diseases. The major advantage of PET/CT is that the localization of the inflammation can be determined. As most patients with MAC-PD after antimycobacterial therapy could have post-inflammatory pulmonary nodules, it allows the differentiation of active lesions from inactive ones.

However, culture-negative conversions were not associated with any of these indicators. This outcome could be attributed to two factors. First, one patient with deteriorating imaging results and symptoms was unable to produce sputum of sufficient quality, resulting in a negative culture. Second, another patient was unable to expectorate sputum. In recent years, patient-reported outcomes (PROs) have gained prominence over microbiological examinations for treatment evaluation [18]. In this study, CAT, a relatively simple evaluation method, was utilized as PROs. A correlation was observed between the pre- and post-treatment ratio of CAT and the clinical treatment effect. However, the pre-treatment CAT score did not demonstrate utility in predicting the treatment response. Furthermore, no correlation was found between pre-treatment CAT and any of the initial PET/CT findings. Thus, PRO can be useful in determining treatment response. Nevertheless, PRO cannot be elucidated solely by the FDG uptake, i.e., the degree of local inflammation.

Although FDG-PET/CT was shown to be useful for determining treatment efficacy in this study, several issues need to be addressed in actual practice. First, the cost of the test is high. Second, it is difficult to evaluate the response to treatment using a single test and requires a baseline test because of the large variation in the SUV peak and other values among patients. In addition, FDG uptake markers at pre-treatment did not reflect the clinical response to treatment. Third, PET/CT findings can be affected by complications arising from other respiratory infections and should be evaluated with caution in cases where such complications are present.

One of the major clinical challenges associated with MAC-PD is the treatment duration. Although treatment for 15 months after culture-negative patients has been shown to have a low relapse rate [19], the duration of treatment remains controversial. The confirmation of sputum culture-negative conversion is impeded in certain cases because of the inability to obtain sputum samples, thereby preventing the execution of culture tests. Moreover, the treatment success varies depending on the patient's immune status. Case 8, which exhibited no recurrence, demonstrated minimal accumulation (SUV peak=1.01) at the time of treatment cessation. The magnitude of the SUV peak may serve as a valuable indicator for determining the appropriate timing of treatment cessation. The utility of FDG-PET/CT as a diagnostic tool for determining treatment discontinuation warrants further investigation. Furthermore, given that certain patients with MAC-PD present as asymptomatic, it may be beneficial to utilize FDG-PET/CT for the assessment of inflammation as a criterion for initiating treatment.

A notable strength of this study was its prospective design, with evaluations conducted on a predetermined schedule.

However, this study has several limitations. First, this study has a single-center design and a small sample size. Second, we defined the culture-negative conversion as at least one negative confirmation at the time of evaluation due to feasibility in an outpatient practice. This could overestimate the culture's negative conversion rate. Furthermore, the study population did not include immunocompromised patients or those with the fibrocavitary type, which is associated with poor prognosis.

## Conclusions

Our results suggest that FDG-PET/CT is valuable for evaluating disease activity in patients with MAC-PD. Given the limitations of this pilot study, including a restricted sample size, it is advisable to conduct large-scale multicenter prospective studies in the future. Additionally, clinical trials investigating the utility of FDG-PET/CT in determining the optimal timing for treatment cessation are warranted.

## Appendices

Appendix A

PET/CT Protocol

18F-FDG PET/CT was performed by Siemens mCT, Germany, or Cartesion Prime PCD-1000A, Canon Medical Systems, Japan. All patients were fasted for at least six hours before the examination. A total of 146 to 308 MBq of FDG was administered intravenously. After one hour of injections, imaging was performed from the head to the knee. Imaging analysis SUV max, SUV peak, TBL, MTV, and TLG were evaluated as semi-quantitative indices of volume accumulation; SUV peak is defined as the average SUV within a small, fixed-size region of interest centered on a highest uptake, the threshold for MTV was set at SUV max 2.5, TLG was calculated as SUV mean × MTV in the lesion, and TBL was calculated as peak in the lesion/mean in the normal lung. Metavol, an open-source software, was used for analysis. When the threshold is set to 2.5, regions exceeding SUV max 2.5 are represented in blue. Lung lesions with an accumulation above the threshold are selected and colored red. The SUV max, SUV peak, MTV, and TLG of the colored lesions are automatically measured. Once the ROI is set on the lesion, the SUV max is automatically calculated.

## References

[1] Morimoto K, Hasegawa N, Izumi K (2017). A laboratory-based analysis of nontuberculous mycobacterial lung disease in Japan from 2012 to 2013. Ann Am Thorac Soc.

[2] Namkoong H, Kurashima A, Morimoto K, Hoshino Y, Hasegawa N, Ato M, Mitarai S (2016). Epidemiology of pulmonary Nontuberculous Mycobacterial Disease, Japan. Emerg Infect Dis.

[3] Harada K, Hagiya H, Funahashi T, Koyama T, Kano MR, Otsuka F (2021). Trends in the nontuberculous mycobacterial disease mortality rate in Japan: a nationwide observational study, 1997-2016. Clin Infect Dis.

[4] Daley CL, Iaccarino JM, Lange C (2020). Treatment of nontuberculous mycobacterial pulmonary disease: an official ATS/ERS/ESCMID/IDSA clinical practice guideline. Eur Respir J.

[5] Kung BT, Seraj SM, Zadeh MZ (2019). An update on the role of (18)F-FDG-PET/CT in major infectious and inflammatory diseases. Am J Nucl Med Mol Imaging.

[6] Barrington SF, Mikhaeel NG, Kostakoglu L (2014). Role of imaging in the staging and response assessment of lymphoma: consensus of the International Conference on Malignant Lymphomas Imaging Working Group. J Clin Oncol.

[7] Cheson BD, Fisher RI, Barrington SF, Cavalli F, Schwartz LH, Zucca E, Lister TA (2014). Recommendations for initial evaluation, staging, and response assessment of Hodgkin and non-Hodgkin lymphoma: the Lugano classification. J Clin Oncol.

[8] Via LE, Schimel D, Weiner DM (2012). Infection dynamics and response to chemotherapy in a rabbit model of tuberculosis using [¹⁸F]2-fluoro-deoxy-D-glucose positron emission tomography and computed tomography. Antimicrob Agents Chemother.

[9] Uruga H, Ishihara M, Hanada S (2014). Evaluation of mycobacterial infections using 18F-fluorodeoxyglucose-positron emission tomography: results of nine cases (Article in Japanese). Kekkaku.

[10] Baxter CG, Bishop P, Low SE, Baiden-Amissah K, Denning DW (2011). Pulmonary aspergillosis: an alternative diagnosis to lung cancer after positive [18F]FDG positron emission tomography. Thorax.

[11] Demura Y, Tsuchida T, Uesaka D (2009). Usefulness of 18F-fluorodeoxyglucose positron emission tomography for diagnosing disease activity and monitoring therapeutic response in patients with pulmonary mycobacteriosis. Eur J Nucl Med Mol Imaging.

[12] Chen RY, Dodd LE, Lee M (2014). PET/CT imaging correlates with treatment outcome in patients with multidrug-resistant tuberculosis. Sci Transl Med.

[13] Chen D, Chen Y, Yang S (2024). The additional value of (18)F-FDG PET/CT imaging in guiding the treatment strategy of non-tuberculous mycobacterial patients. Respir Res.

[14] World Medical Association (2013). World Medical Association Declaration of Helsinki: ethical principles for medical research involving human subjects. JAMA.

[15] Griffith DE, Aksamit T, Brown-Elliott BA (2007). An official ATS/IDSA statement: diagnosis, treatment, and prevention of nontuberculous mycobacterial diseases. Am J Respir Crit Care Med.

[16] Kohno S, Aoki N, Kadota J (2012). Clinical evaluation of new antimicrobial agents in respiratory infections (2nd ed.). Jpn J Chemother.

[17] Sehgal IS, Arora K, Agarwal R (2025). Role of serial fluorodeoxyglucose positron emission tomography-computed tomography (18FDG-PET-CT) in assessing treatment response in treatment naive chronic pulmonary aspergillosis subjects. J Infect Dis.

[18] Flume PA, Griffith DE, Chalmers JD (2021). Development of drugs for nontuberculous mycobacterial disease: clinicians’ interpretation of a US Food and Drug Administration workshop. Chest.

[19] Furuuchi K, Morimoto K, Kurashima A (2020). Treatment duration and disease recurrence following the successful treatment of patients with Mycobacterium avium complex lung disease. Chest.

